# Cystitis Caused by Citrobacter amalonaticus in an Older Outpatient in the Absence of Catheterization

**DOI:** 10.7759/cureus.109520

**Published:** 2026-05-23

**Authors:** Yoshihiro Kawaguchi

**Affiliations:** 1 Department of Urology, Morodomi Central Hospital, Saga, JPN

**Keywords:** citrobacter amalonaticus, older adults, postvoid residual urine, urinary tract infections, uropathogens

## Abstract

*Citrobacter amalonaticus *(*C. amalonaticus*) is a rarely isolated member of the *Citrobacter* genus and an uncommon cause of human infection. Although *Citrobacter* species are recognized uropathogens, the clinical significance of *C. amalonaticus* in urinary tract infections (UTIs) remains poorly characterized.

We report a case of cystitis caused by *C. amalonaticus* in an 87-year-old woman who visited our outpatient clinic owing to urinary symptoms. She had previously received medication for an overactive bladder and had persistent postvoid residual urine. Urine culturing yielded only *C. amalonaticus*, and the patient was successfully treated with levofloxacin.

Reports of cystitis caused by *C. amalonaticus* in non-catheterized outpatients are extremely limited. In older patients, urinary tract infections are often associated with comorbidities and impaired bladder emptying, which may predispose them to infections by uncommon organisms.

This case highlights the fact that *C. amalonaticus* can act as a uropathogen in older patients with urinary stasis, even in the absence of catheterization. Clinicians should be aware that this rare organism is a potential cause of urinary tract infections.

## Introduction

*Citrobacter* species are opportunistic Gram-negative bacilli belonging to the order Enterobacterales and are known to cause various infections, including urinary tract infections (UTIs), particularly in vulnerable populations. Among them, *Citrobacter amalonaticus* (*C. amalonaticus*) is a rarely isolated species that is infrequently identified in clinical specimens compared with more common species such as *C. freundii* and *C. koseri*. Recent advances in automated identification systems and matrix-assisted laser desorption/ionization time-of-flight mass spectrometry (MALDI-TOF MS) have improved the recognition of this uncommon organism in clinical practice. However, only a limited number of human infections caused by *C. amalonaticus* have been reported [1], and its clinical significance as a uropathogen remains poorly characterized [2].

UTIs are common in older patients and are often associated with multiple predisposing factors, including comorbidities and functional urinary abnormalities such as impaired bladder emptying [3]. These conditions may predispose susceptible individuals to infections caused by uncommon organisms. Most previously reported *C. amalonaticus* infections involved hospitalized, catheterized, or immunocompromised patients. Herein, we report a rare case of community-acquired cystitis caused by *C. amalonaticus* in a non-catheterized older outpatient with persistent postvoid residual urine.

## Case presentation

An 87-year-old woman presented to our outpatient clinic with dysuria and urinary frequency. She was afebrile and had no flank pain, chills, gross hematuria, or other systemic symptoms. Urinalysis revealed pyuria (3+) and turbid urine, and she was diagnosed with cystitis. Her medical history included chronic heart failure and chronic kidney disease. Three years previously, she had experienced cystitis caused by *Escherichia coli*.

Two years before presentation, she had been diagnosed with an overactive bladder and treated with solifenacin, an antimuscarinic agent, and vibegron, a β3-adrenoceptor agonist. Her postvoid residual urine volume, measured by transabdominal ultrasonography, had been approximately 114 mL.

Urine culture was performed, and oral levofloxacin 500 mg/day was administered for five days, resulting in the prompt resolution of her symptoms. Urine culture yielded >107 colony-forming units (CFU)/mL of *C. amalonaticus* with 4+ growth as a single isolate. The organism was identified using matrix-assisted laser desorption/ionization time-of-flight mass spectrometry (MALDI-TOF MS). Antimicrobial susceptibility testing was performed using the broth microdilution method and demonstrated susceptibility to levofloxacin (Table 1).

**Table 1:** Antimicrobial susceptibility profile of Citrobacter amalonaticus isolated from urine culture. Antimicrobial susceptibility testing demonstrated that the isolate was susceptible to levofloxacin, which was selected for treatment. ABPC, ampicillin; T/P, tazobactam/piperacillin; PIPC, piperacillin; S/A, sulbactam/ampicillin; CEZ, cefazolin; CMZ, cefmetazole; CAZ, ceftazidime; CTRX, ceftriaxone; CPDX, cefpodoxime; FMOX, flomoxef; S/C, sulbactam/cefoperazone; CFPN, cefcapene; MINO, minocycline; GM, gentamicin; AMK, amikacin; CP, chloramphenicol; AZT, aztreonam; ST, sulfamethoxazole/trimethoprim; FRPM, faropenem; IPM, imipenem; MEPM, meropenem; CPFX, ciprofloxacin; LVFX, levofloxacin; FOM, fosfomycin; MIC, minimum inhibitory concentration; S, susceptible; R, resistant

Antibiotic	Interpretation	MIC (μg/mL)
ABPC	R	>16
T/P	S	≦8
PIPC	S	≦8
S/A	S	≦8
CEZ	R	>16
CMZ	S	≦4
CAZ	S	≦1
CTRX	S	≦1
CPDX	S	≦1
FMOX	S	≦8
S/C	S	≦16
CFPN	S	≦1
MINO	S	≦1
GM	S	≦2
AMK	S	≦4
CP	S	≦8
AZT	S	≦1
ST	S	≦40
FRPM	S	≦1
IPM	S	≦1
MEPM	S	≦0.12
CPFX	S	≦0.06
LVFX	S	≦0.12
FOM	S	≦64

At a follow-up visit one month later, her postvoid residual urine volume remained approximately 110 mL. Therefore, solifenacin was discontinued, and vibegron therapy was continued. No recurrence of urinary symptoms was observed during the following month.

## Discussion

This case provides two important clinical insights. First, *C. amalonaticus* can act as a causative pathogen of cystitis in older patients with predisposing factors such as urinary stasis. Second, infections caused by this organism can occur even in non-catheterized outpatient settings.

*Citrobacter amalonaticus* is a rarely reported opportunistic pathogen in humans, and UTI due to this organism remains uncommon. Most available clinical data are derived from isolated case reports and small case series. Garcia et al. reported that *C. amalonaticus* accounts for a small proportion of *Citrobacter*-associated UTIs, predominantly affecting elderly or comorbid patients, often with underlying urological abnormalities or healthcare exposure [1]. Our case is consistent with these observations, as the patient was an elderly woman with chronic heart failure, chronic kidney disease, and persistent postvoid residual urine.

In the present case, the patient demonstrated a persistent residual urine volume of approximately 110-114 mL despite treatment for overactive bladder with both an antimuscarinic agent and a β3-adrenoceptor agonist. This finding suggests that incomplete bladder emptying may have contributed to urinary stasis and subsequent infection. In elderly patients, postvoid residual urine is a well-established risk factor for recurrent UTI due to bacterial proliferation in stagnant urine [3]. Moreover, antimuscarinic agents may exacerbate voiding dysfunction, particularly in frail older adults, thereby increasing susceptibility to infection.

From a microbiological perspective, *C. amalonaticus* is an Enterobacterales species closely related to other *Citrobacter* species, but it is infrequently isolated in routine clinical practice. Reported infections include bacteremia, peritoneal dialysis-associated peritonitis, and device-related infections [4-6]. Although urinary tract infection is the most common clinical manifestation, it remains rare compared with *E. coli*-associated UTI. The increasing recognition of this organism may be partly attributable to the widespread use of advanced identification techniques such as MALDI-TOF MS, which improves discrimination among *Citrobacter* species that were previously misidentified [1].

Antimicrobial susceptibility patterns of *C. amalonaticus* remain incompletely characterized due to the limited number of reported cases. However, multidrug-resistant strains, including carbapenem-resistant isolates, have been described [5]. In contrast, susceptibility to fluoroquinolones is often preserved. In the present case, the isolate was susceptible to levofloxacin, and clinical resolution was achieved promptly with oral therapy. Table 2 summarizes previously reported cases of *C. amalonaticus* infection, including infection site, host characteristics, antimicrobial susceptibility, and clinical outcomes.

**Table 2:** Summary of previously reported cases of Citrobacter amalonaticus infection. This table summarizes previously reported cases of *C. amalonaticus* infection, including infection site, patient characteristics, underlying conditions, microbiological identification methods, antimicrobial susceptibility profiles, treatment regimens, and clinical outcomes. This overview highlights the rarity of urinary tract infection (UTI) cases, particularly in non-catheterized patients. PVR, postvoid residual; NA, not available

Author	Year	Infection type	Patient background	Catheter	Outcome
Garcia et al. [1]	2016	UTI	Older/immunocompromised	Mixed	Improved
Wong et al. [6]	2012	Peritonitis	Peritoneal dialysis patient	NA	Improved
Lai et al. [4]	2010	Bacteremia	Hospitalized	NA	Variable
Midlick et al. [5]	2024	Bacteremia after Rezum	Post-urological procedure	Yes	Improved
Kawaguchi (present case)	2026	Cystitis	Older outpatient with PVR	No	Improved

UTIs in older adults are influenced by multiple factors, including immunosenescence, comorbidities, and functional urinary tract changes. Rodriguez-Mañas emphasized that UTIs in elderly patients often present with mild or atypical symptoms and are frequently associated with underlying structural or functional abnormalities of the urinary tract [3]. The absence of systemic symptoms in our patient is consistent with this clinical pattern, and the favorable response to oral antimicrobial therapy highlights the importance of early diagnosis and targeted treatment in this population.

From a management perspective, the reduction of pharmacologically induced voiding dysfunction may be beneficial in patients with significant postvoid residual urine. In this case, the discontinuation of solifenacin while continuing vibegron resulted in stable residual urine volume and no recurrence of symptoms during short-term follow-up. This suggests that minimizing antimuscarinic-induced urinary retention may help reduce the risk of recurrent infection.

Finally, this case adds to the limited literature on *C. amalonaticus* infections and reinforces several clinically relevant points summarized in Table 2.

In addition to UTI, this organism has been implicated in bloodstream infections, peritonitis, and post-procedural infections in both immunocompromised and immunocompetent hosts [4-7]. Taken together, these findings underscore the need to consider uncommon Enterobacterales species in elderly patients with urinary stasis and highlight the diagnostic utility of MALDI-TOF MS in accurate pathogen identification. Further accumulation of cases is necessary to clarify the epidemiology, resistance mechanisms, and optimal management strategies for this rare uropathogen.

## Conclusions

*Citrobacter amalonaticus* is a rare but clinically relevant cause of UTI in elderly patients with urinary stasis, and it can occur even in non-catheterized outpatients. Accurate identification by MALDI-TOF MS is important because this organism may be underrecognized among *Citrobacter* species. This case also suggests that reducing antimuscarinic-induced urinary retention may help prevent recurrence. Further case accumulation is needed to clarify its clinical characteristics and optimal management.
